# Supplementary material for: The differential impact of climate interventions along the political divide in 60 countries
Source: Nat Commun. 2024 May 8;15:3885. doi: 10.1038/s41467-024-48112-8 (PMC11078920; doi:10.1038/s41467-024-48112-8)
Supplement: Supplementary file 1 — Supplementary Information [file 41467_2024_48112_MOESM1_ESM.pdf]

Supplementary Materials

The Differential Impact of Climate Interventions along the Political Divide in 60 Countries

Michael Berkebile-Weinberg[1,†], Danielle Goldwert[1,†], Kimberly C. Doell[2], Jay J. Van Bavel[1,3], & Madalina Vlasceanu[1,*]

[1]Department of Psychology, New York University
[2]Department of Cognition, Emotion, and Methods in Psychology, Faculty of Psychology, University of Vienna
[3]Norwegian School of Economics

† These authors contributed equally
* Address correspondence to Madalina Vlasceanu [vlasceanu@nyu.edu]

Data and materials availability

All data (including sample information per country) can be found on OSF: https://osf.io/ytf89/ and all code can be found on GitHub: https://github.com/mvlasceanu/climatepolarization. DOI versions of these links can be found in the main article text.

Supplementary Figures

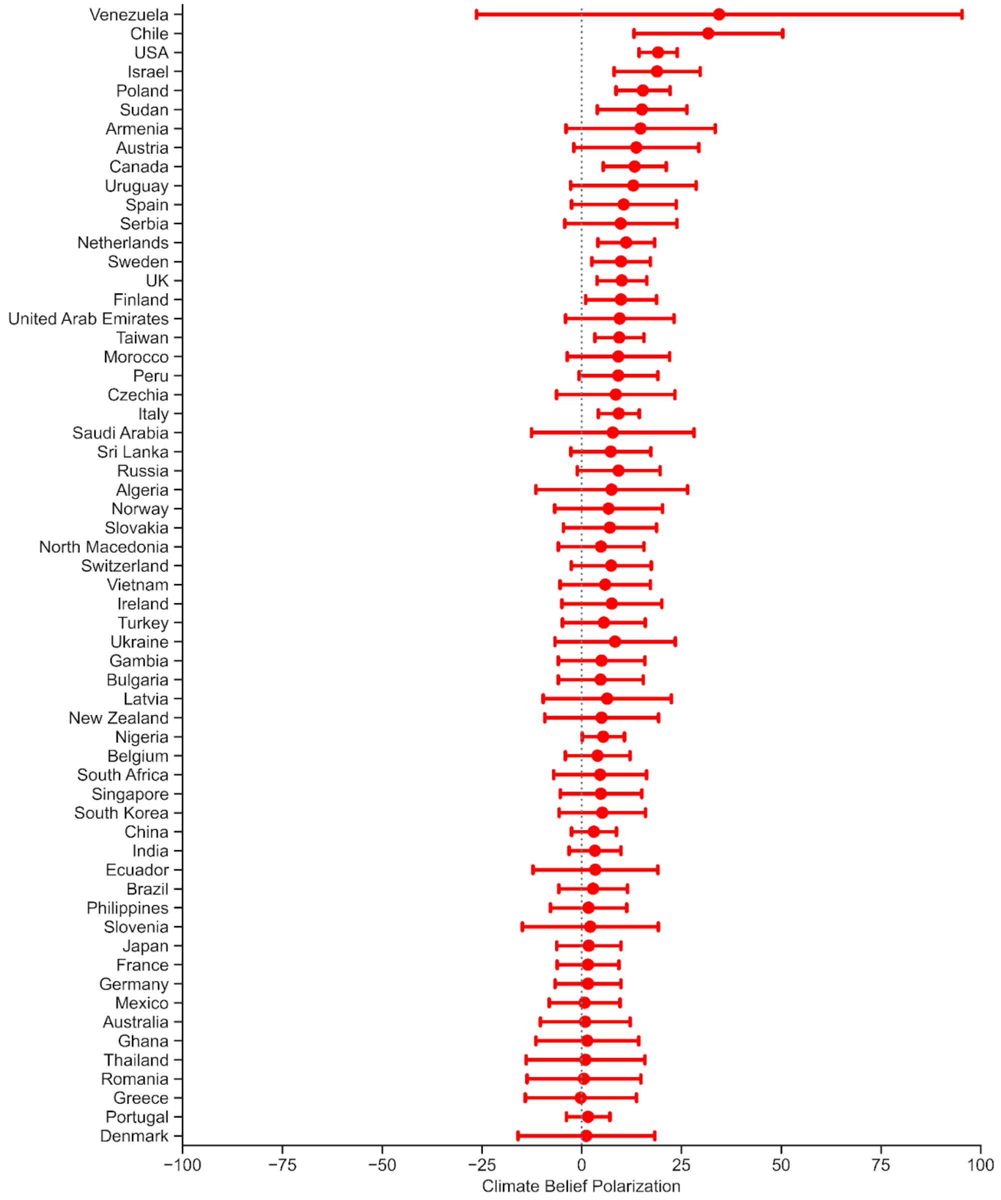

Fig S1. Means and 95% confidence intervals around the means of the degree of belief polarization, defined as the absolute value of the difference between self-reported liberals' and conservatives' belief in climate change.

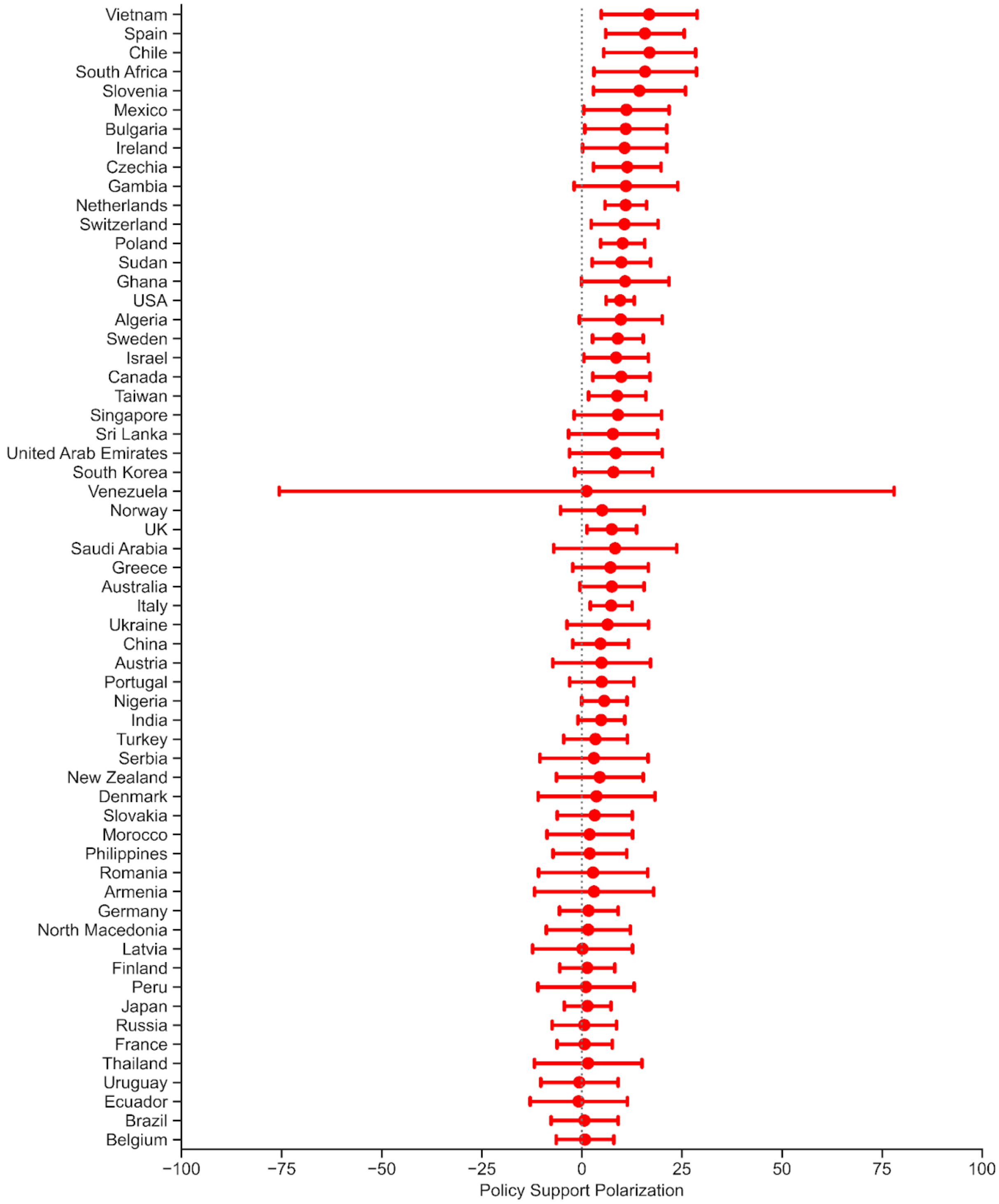

Fig S2. Means and 95% confidence intervals around the means of the degree of climate policy support polarization, defined as the absolute value of the difference between self-reported liberals' and conservatives' climate policy support.

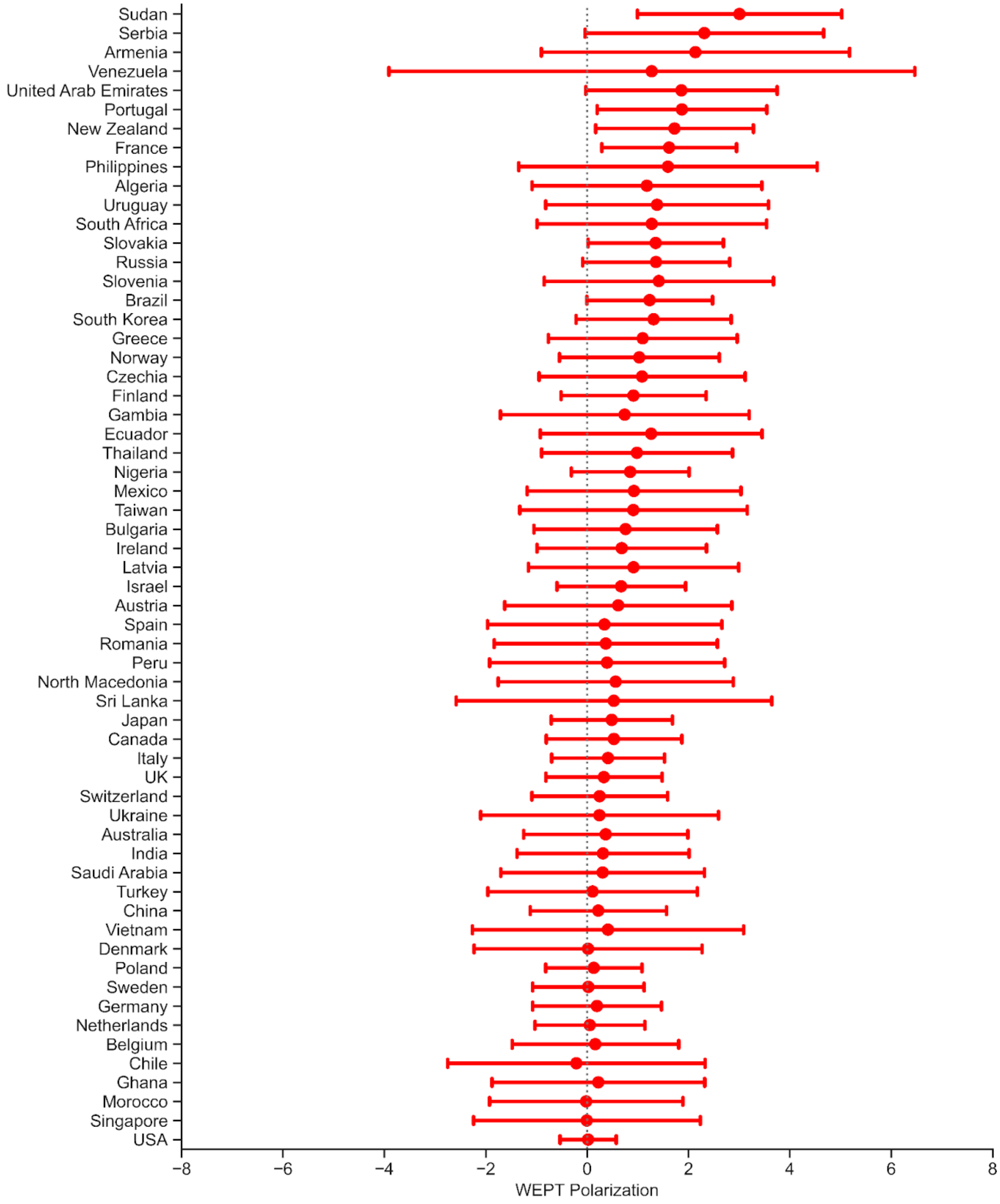

Fig S3. Means and 95% confidence intervals around the means of the degree of climate action polarization, defined as the absolute value of the difference between self-reported liberals' and conservatives' climate action.

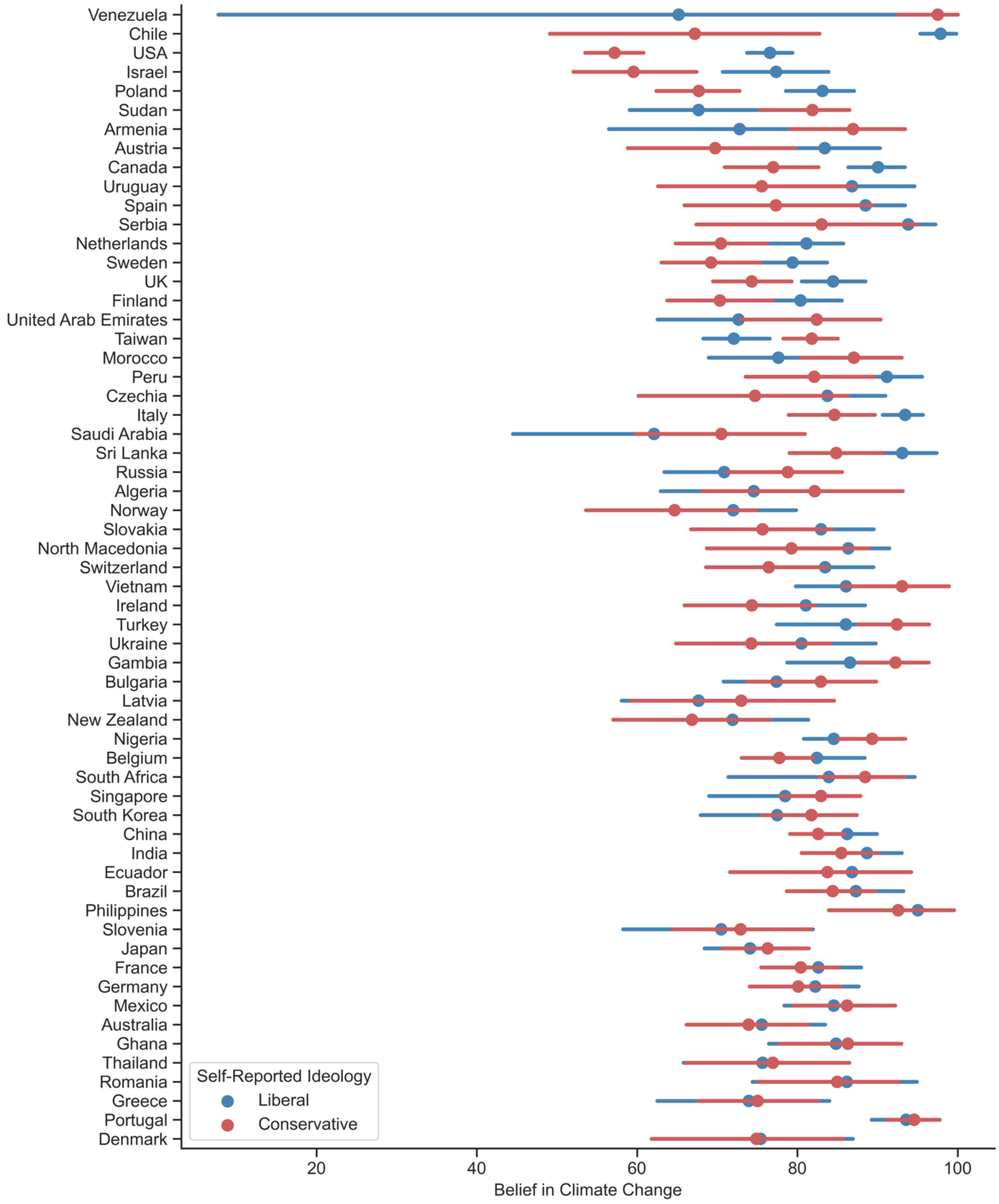

Fig S4. Means and 95% confidence intervals around the means of the belief in climate change raw scores of self-reported liberals (in blue) and conservatives (in red), by country.

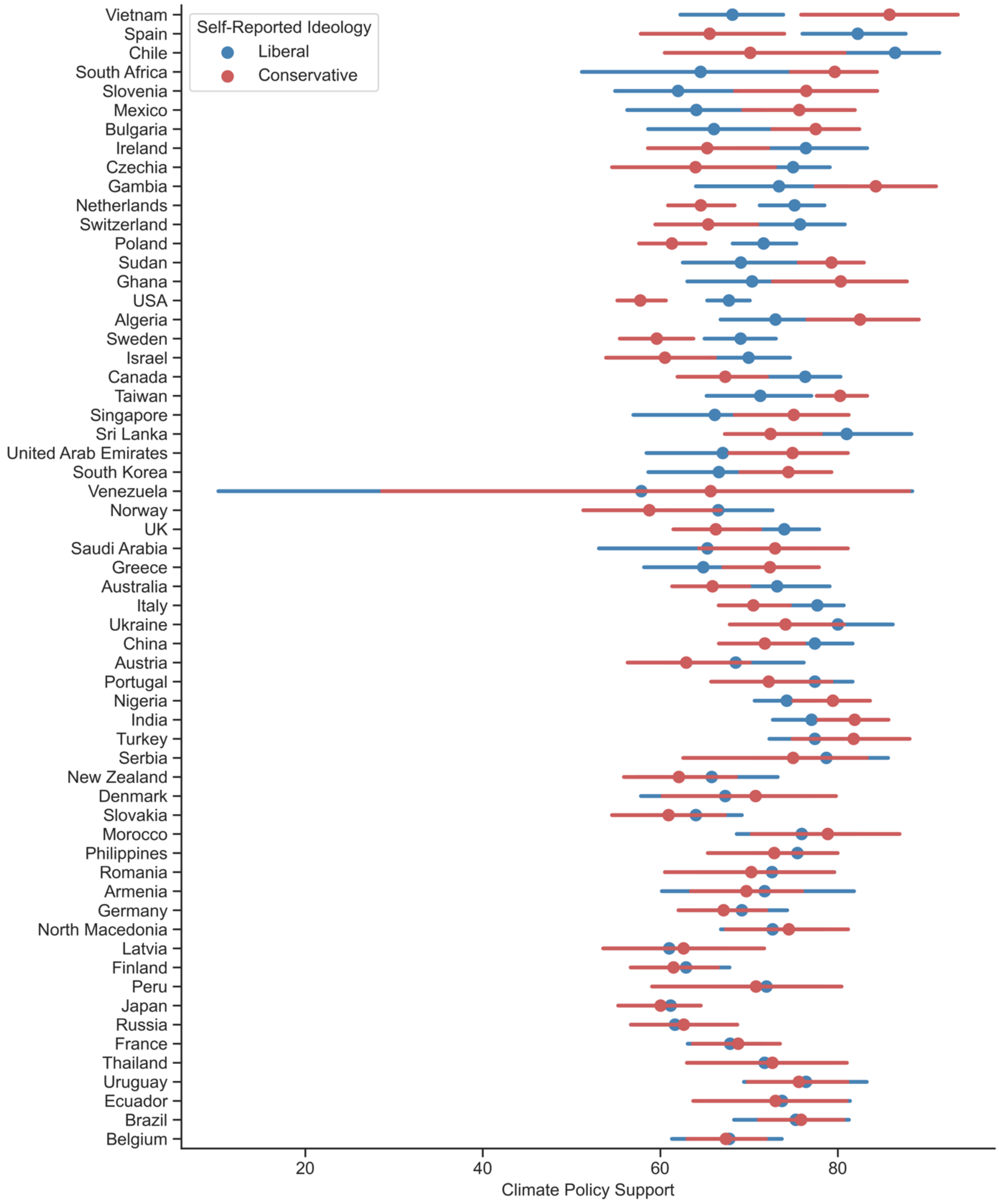

Fig S5. Means and 95% confidence intervals around the means of the climate policy support raw scores of self-reported liberals (in blue) and conservatives (in red), by country.

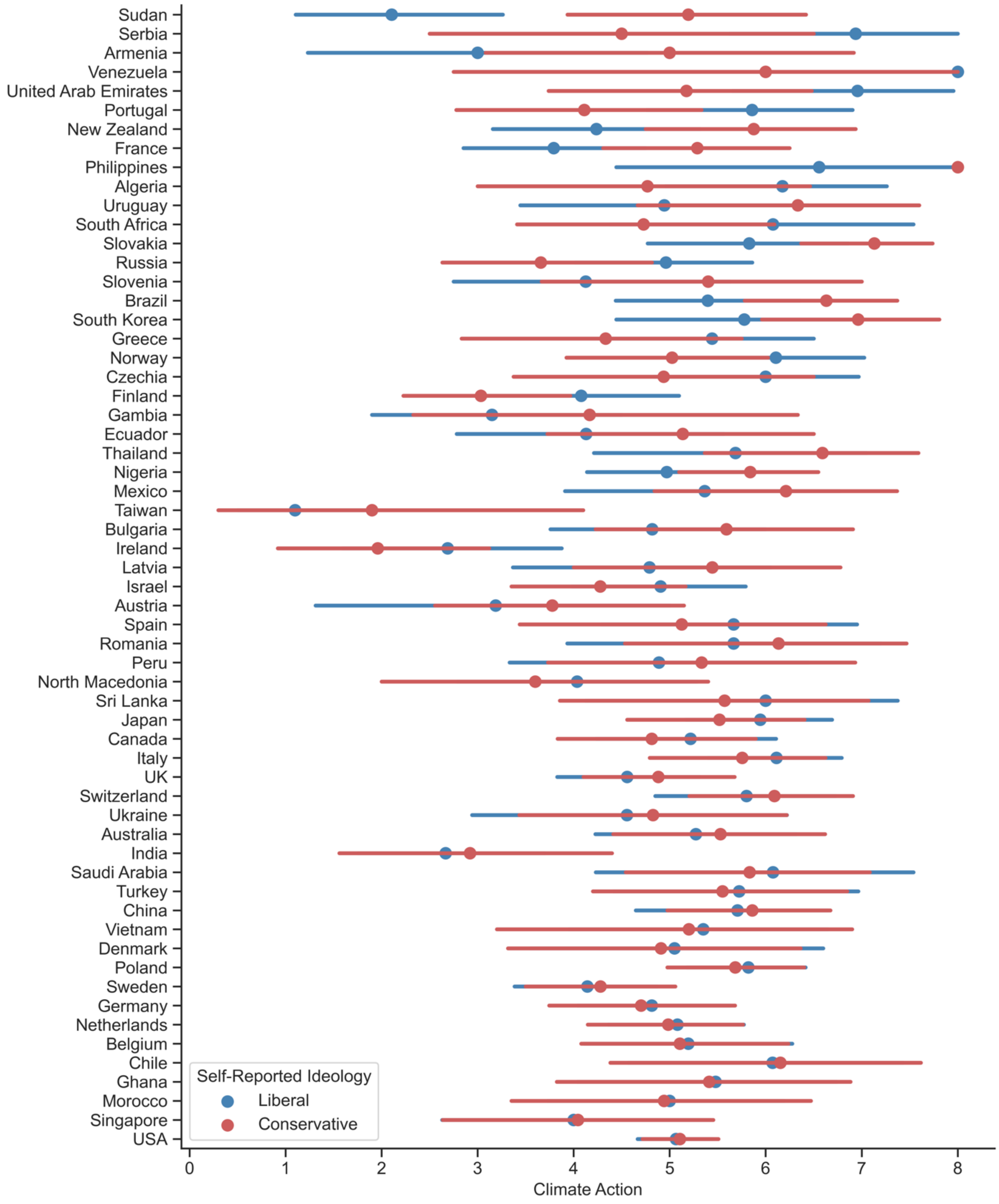

Fig S6. Means and 95% confidence intervals around the means of the climate action raw scores of self-reported liberals (in blue) and conservatives (in red), by country.

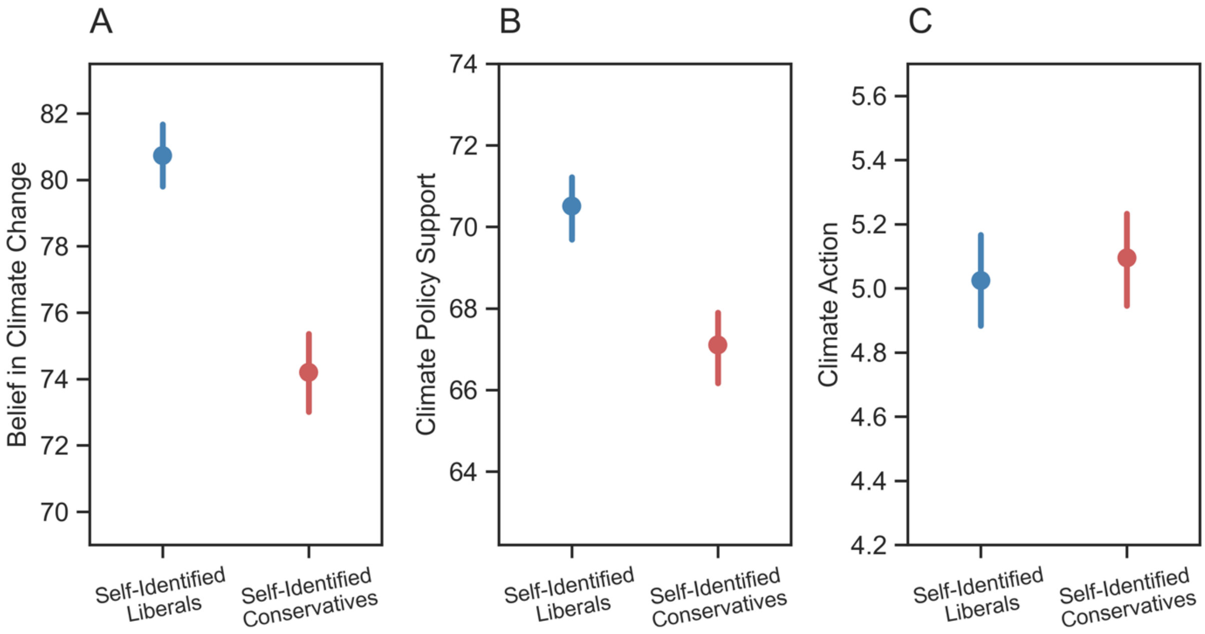

Fig S7. Belief in climate change (Panel A), climate policy support (Panel B), and climate action (Panel C) as a function of participants' self-reported political ideology (liberals versus conservatives), computed as a median split within country of the continuous ideology measure.

	Climate Beliefs		Climate Policy Support		Climate Action	
	Self-ID Liberals	Self-ID Conservatives	Self-ID Liberals	Self-ID Conservatives	Self-ID Liberals	Self-ID Conservatives
Psych. Distance	✓	✓	✓		✗	✗
Letter to Future Gen.		✓	✓	✓	✗	✗
Collective Action	✓	✓	✓	✓		✗
Future-Self Cont.	✓	✓	✓	✓		✗
Neg. Emotions				✗	✗	✗
System Justification	✓	✓				✗
Sci. Consensus					✓	
Binding Moral Found.						
Dynamic Norms						
Pluralistic Ignorance						✗
Work Together Norm					✗	✗

Figure S8. Results of linear mixed effects models with climate outcomes (climate beliefs, policy support, and action) as the dependent variable, condition (11 interventions versus control) as it interacts with self-reported political ideology (here split by midpoint on ideology scales) as the fixed effects, including by-participant and by-country random effects. The green check marks indicate statistically significant increases compared to control, and the red X marks indicate statistically significant decreases compared to control, and empty cells indicate no statistically significant differences compared to control.

1. Supplementary Tables

Table S1. Interventions' effects on self-identified liberals' climate beliefs, compared to the control condition. Analysis was conducted using only self-identified liberals' data (using a per-country median split of the continuous ideology measure). Statistical analysis was performed using a linear mixed effects model with condition as a fixed effect, and by-participant, by-country, and by-item (i.e., 4 beliefs) random effects. Tests on fixed effects are two-tailed, and no adjustments for multiple comparisons were made.

Intervention	Estimate	*SE*	*df*	*t*	*p*	95% CI	Cohen's *d*
(Intercept)	81.32	1.00	74.68	81.22	< .001	[79.33, 83.31]	18.80
PsychDistance	2.79	0.67	25343.92	4.17	< .001	[1.48, 4.10]	0.05
FutureSelfCont	2.12	0.69	25341.28	3.08	.002	[0.77, 3.47]	0.04
SystemJust	1.91	0.65	25339.41	2.95	.003	[0.64, 3.18]	0.04
CollectAction	1.80	0.65	25336.96	2.76	.006	[0.53, 3.07]	0.03
BindingMoral	1.39	0.65	25341.90	2.13	.033	[0.12, 2.66]	0.03
LetterFutureGen	1.47	0.70	25346.89	2.11	.035	[0.10, 2.84]	0.03
SciConsens	1.22	0.65	25337.74	1.88	.060	[-0.05, 2.49]	0.02
PluralIgnorance	0.67	0.65	25336.83	1.03	.305	[-0.60, 1.94]	0.01
NegativeEmotions	0.59	0.64	25336.42	0.92	.358	[-0.66, 1.84]	0.01
DynamicNorm	0.55	0.65	25339.98	0.85	.394	[-0.72, 1.82]	0.01
WorkTogetherNorm	-0.72	0.65	25338.41	-1.10	.270	[-1.99, 0.55]	0.01

Table S2. Interventions' effects on self-identified conservatives' climate beliefs, compared to the control condition. Analysis was conducted using only self-identified conservatives' data (using a per-country median split of the continuous ideology measure). Statistical analysis was performed using a linear mixed effects model with condition as a fixed effect, and by-participant, by-country, and by-item (i.e., 4 beliefs) random effects. Tests on fixed effects are two-tailed, and no adjustments for multiple comparisons were made.

Intervention	Estimate	*SE*	*df*	*t*	*p*	*95% C.I.*	Cohen's *d*
(Intercept)	78.44	1.11	99.89	70.93	< .001	[76.23, 80.65]	14.19
PsychDistance	3.52	0.76	24331.56	4.61	< .001	[2.03, 5.01]	0.06
CollectAction	2.58	0.75	24336.05	3.45	< .001	[1.11, 4.05]	0.04
LetterFutureGen	2.44	0.80	24331.44	3.05	.002	[0.87, 4.01]	0.04
FutureSelfCont	1.58	0.79	24332.33	2.00	.045	[0.032, 3.13]	0.03
SystemJust	1.48	0.75	24333.48	1.98	.048	[0.01, 2.95]	0.03
SciConsens	1.12	0.74	24332.59	1.51	.132	[-0.33, 2.57]	0.02
BindingMoral	0.92	0.75	24332.56	1.23	.218	[-0.55, 2.39]	0.02
NegativeEmotions	0.52	0.76	24330.14	0.68	.495	[-0.97, 2.00]	< 0.001
PluralIgnorance	0.51	0.74	24329.44	0.68	.494	[-0.94, 1.96]	< 0.001
DynamicNorm	0.36	0.75	24337.24	0.49	.628	[-1.11, 1.83]	< 0.001
WorkTogetherNorm	-0.33	0.75	24329.61	-0.44	.657	[-1.8, 1.14]	< 0.001

Table S3. Interventions' effects on self-identified liberals' climate policy support, compared to the control condition. Analysis was conducted using only self-identified liberals' data (using a per-country median split of the continuous ideology measure). Statistical analysis was performed using a linear mixed effects model with condition as a fixed effect, and by-participant, by-country, and by-item (i.e., 9 policies) random effects. Tests on fixed effects are two-tailed, and no adjustments for multiple comparisons were made.

Intervention	Estimate	*SE*	*df*	*t*	*p*	95% *C.I.*	Cohen's *d*
(Intercept)	70.84	4.15	8.56	17.06	<.001	[62.58, 79.10]	11.66
CollectiveAction	3.01	0.56	25156.57	5.40	<.001	[1.91, 4.11]	0.07
LetterFuture	2.59	0.60	25179.85	4.34	<.001	[1.41, 3.77]	0.05
FutureSelfCont	1.95	0.59	25182.39	3.31	.001	[0.79, 3.11]	0.04
PsychDistance	1.73	0.57	25204.65	3.02	.003	[0.61, 2.85]	0.04
SystemJust	1.34	0.55	25173.50	2.42	.015	[0.26, 2.42]	0.03
DynamicNorm	0.77	0.55	25180.65	1.40	.163	[-0.31, 1.85]	0.02
BindingMoral	0.66	0.56	25167.53	1.19	.234	[-0.44, 1.76]	0.01
SciConsensus	0.44	0.55	25188.94	0.79	.428	[-0.64, 1.52]	< 0.001
NegativeEmotions	0.29	0.55	25179.09	0.53	.599	[-0.79, 1.37]	< 0.001
PluralIgnorance	-0.12	0.55	25170.33	-0.21	.834	[-1.20, 0.96]	< 0.001
WorkTogetherNorm	-0.19	0.56	25199.81	-0.34	.733	[-1.29, 0.91]	< 0.001

Table S4. Interventions' effects on self-identified conservatives' climate policy support, compared to the control condition. Analysis was conducted using only self-identified conservatives' data (using a per-country median split of the continuous ideology measure). Statistical analysis was performed using a linear mixed effects model with condition as a fixed effect, and by-participant, by-country, and by-item (i.e., 9 policies) random effects. Tests are two-tailed, and no adjustments for multiple comparisons were made.

Intervention	Estimate	*SE*	*df*	*t*	*p*	95% *C.I.*	Cohen's *d*
(Intercept)	70.07	4.07	8.93	17.23	<.001	[60.86, 79.28]	11.53
LetterFuture	2.83	0.63	24225.55	4.51	<.001	[1.60, 4.06]	0.06
CollectiveAction	2.65	0.59	24212.56	4.51	<.001	[1.49, 3.80]	0.06
FutureSelfCont	1.60	0.62	24225.32	2.58	.010	[0.38, 2.82]	0.03
SciConsensus	0.91	0.58	24240.60	1.56	.120	[-0.23, 2.05]	0.02
PsychDistance	1.04	0.60	24246.45	1.73	.084	[-0.14, 2.21]	0.02
DynamicNorm	0.63	0.59	24233.66	1.08	.282	[-0.52, 1.79]	0.01
BindingMoral	0.55	0.59	24218.82	0.93	.355	[-0.61, 1.71]	0.01
PluralIgnorance	0.36	0.58	24221.30	0.61	.542	[-0.78, 1.50]	0.01
SystemJust	0.37	0.59	24221.61	0.62	.536	[-0.79, 1.53]	0.01
WorkTogetherNorm	-0.21	0.59	24225.40	-0.35	.727	[-1.37, 0.95]	-0.004
NegativeEmotions	-1.22	0.60	24208.00	-2.04	.041	[-2.40, -0.04]	-0.03

Table S5. Interventions' effects on self-identified liberals' actions on tree planting task, compared to the control condition. Analysis was conducted using only self-identified liberals' data (using a per-country median split of the continuous ideology measure). Statistical analysis was performed using an ordinal mixed model (i.e., cumulative link mixed model fitted with the Laplace approximation) with condition as a fixed effect, and by-country random effects. Tests are two-tailed, and no adjustments for multiple comparisons were made.

Intervention	Estimate	*SE*	*z*	*p*	95% *C.I.*	Cohen's *d*
SciConsensus	0.12	0.06	2.05	.040	[0.002, 0.24]	0.06
BindingMoral	0.10	0.06	1.77	.077	[-0.02, 0.22]	0.06
DynamicNorm	0.10	0.06	1.72	.085	[-0.02, 0.22]	0.05
SystemJust	0.02	0.06	0.36	.717	[-0.10, 0.14]	0.01
FutureSelfCont	-0.01	0.06	-0.11	.911	[-0.13, 0.11]	-0.00
PluralIgnorance	-0.03	0.06	-0.60	.552	[-0.15, 0.09]	-0.02
CollectiveAction	-0.06	0.06	-1.14	.254	[-0.18, 0.06]	-0.04
WorkTogetherNorm	-0.15	0.06	-2.69	.007	[-0.27, -0.03]	-0.08
LetterFuture	-0.17	0.06	-2.87	.004	[-0.29, -0.05]	-0.10
PsychDistance	-0.21	0.06	-3.68	<.001	[-0.33, -0.09]	-0.12
NegativeEmotions	-0.25	0.06	-4.50	<.001	[-0.37, -0.13]	-0.14

Table S6. Interventions' effects on self-identified conservatives' actions on tree planting task, compared to the control condition. Analysis was conducted using only self-identified conservatives' data (using a per-country median split of the continuous ideology measure). Statistical analysis was performed using an ordinal mixed model (i.e., cumulative link mixed model fitted with the Laplace approximation) with condition as a fixed effect, and by-country random effects. Tests are two-tailed, and no adjustments for multiple comparisons were made.

Intervention	Estimate	*SE*	*z*	*p*	95% *C.I.*	Cohen's *d*
BindingMoral	-0.03	0.06	-0.49	.622	[-0.15, 0.09]	-0.02
SciConsensus	-0.06	0.06	-0.99	.321	[-0.18, 0.06]	-0.03
DynamicNorm	-0.10	0.06	-1.73	.084	[-0.22, 0.02]	-0.06
PluralIgnorance	-0.12	0.06	-2.11	.035	[-0.24, -0.002]	-0.07
SystemJust	-0.15	0.06	-2.52	.012	[-0.27, -0.03]	-0.08
CollectiveAction	-0.15	0.06	-2.49	.013	[-0.27, -0.03]	-0.08
FutureSelfCont	-0.21	0.06	-3.45	<.001	[-0.33, -0.09]	-0.12
LetterFuture	-0.32	0.06	-5.19	<.001	[-0.44, -0.20]	-0.18
NegativeEmotions	-0.35	0.06	-5.98	<.001	[-0.47, -0.23]	-0.20
WorkTogetherNorm	-0.37	0.06	-6.45	<.001	[-0.49, -0.25]	-0.21
PsychDistance	-0.35	0.06	-5.86	<.001	[-0.47, -0.23]	-0.19

Table S7. Results of a linear mixed effects model with beliefs in climate change as the dependent variable, condition (11 interventions versus control) as it interacts with self-reported political ideology (median split within each country) as the fixed effects, including by-participant and by-country random effects.

Fixed Effect	Estimate	*SE*	*df*	*t*	*p*	95% *C.I.*	Cohen's *d*
(Intercept)	76.68	0.92	117.18	83.44	<.001	[74.88, 78.48]	15.43
Ideology	6.35	0.71	49720	8.89	<.001	[4.96, 7.74]	0.08
PsychDistance	3.20	0.73	49720	4.40	<.001	[1.77, 4.63]	0.04
CollectiveAction	2.62	0.71	49730	3.68	<.001	[1.23, 4.01]	0.03
LetterFuture	2.64	0.76	49720	3.46	<.001	[1.15, 4.13]	0.03
FutureSelfCont	1.57	0.75	49720	2.09	.037	[0.10, 3.04]	0.02
SystemJust	1.40	0.72	49730	1.95	.051	[-0.01, 2.81]	0.02
SciConsensus	1.08	0.71	49730	1.52	.130	[-0.31, 2.47]	0.01
BindingMoral	0.87	0.72	49720	1.22	.244	[-0.54, 2.28]	0.01
PluralIgnorance	0.60	0.71	49720	0.85	.396	[-0.79, 1.99]	< 0.001
DynamicNorm	0.41	0.71	49730	0.58	.563	[-0.98, 1.80]	< 0.001
NegativeEmotions	0.38	0.73	49720	0.52	.602	[-1.05, 1.81]	< 0.001
WorkTogetherNorm	-0.26	0.71	49720	-0.36	.717	[-1.65, 1.13]	< 0.001
BindingMoral: Ideology	0.60	1.00	49720	0.60	.551	[-1.36, 2.56]	< 0.001
FutureSelfCont: Ideology	0.59	1.06	49720	0.56	.575	[-1.49, 2.67]	< 0.001
DynamicNorm: Ideology	0.31	1.00	49730	0.31	.756	[-1.65, 2.27]	< 0.001
NegativeEmotions: Ideology	0.24	1.00	49720	0.24	.812	[-1.72, 2.20]	< 0.001
SystemJust: Ideology	0.48	1.00	49720	0.48	.634	[-1.48, 2.44]	< 0.001
SciConsensus: Ideology	0.16	1.00	49720	0.16	.874	[-1.80, 2.12]	< 0.001
PluralIgnorance: Ideology	0.22	1.00	49720	0.22	.822	[-1.74, 2.18]	< 0.001
CollectiveAction: Ideology	-0.70	1.00	49720	-0.69	.488	[-2.66, 1.26]	< 0.001
WorkTogetherNorm: Ideology	-0.44	1.00	49720	-0.44	.658	[-2.40, 1.52]	< 0.001
PsychDistance: Ideology	-0.13	1.03	49720	-0.13	.899	[-2.15, 1.89]	< 0.001
LetterFuture: Ideology	-1.14	1.07	49730	-1.07	.286	[-3.24, 0.96]	< 0.001

Table S8. Results of a linear mixed effects model with climate policy support as the dependent variable, condition (11 interventions versus control) as it interacts with self-reported political ideology (median split within each country) as the fixed effects, including by-participant and by-country random effects. Tests are two-tailed, and no adjustments for multiple comparisons were made.

Fixed Effect	Estimate	*SE*	*df*	*t*	*p*	95% *C.I.*	Cohen's *d*
(Intercept)	68.96	0.76	116.1	90.64	<.001	[67.47, 70.45]	16.83
Ideology	3.36	0.58	50280	5.80	<.001	[2.22, 4.50]	0.05
LetterFuture	3.06	0.62	50270	4.90	<.001	[1.85, 4.28]	0.04
CollectiveAction	2.72	0.58	50230	4.66	<.001	[1.58, 3.86]	0.04
FutureSelfCont	1.61	0.62	50260	2.61	.009	[0.40, 2.83]	0.02
SciConsensus	0.92	0.58	50300	1.58	.114	[-0.22, 2.06]	0.01
PsychDistance	0.87	0.60	50310	1.47	.143	[-0.31, 2.05]	0.01
DynamicNorm	0.75	0.58	50280	1.29	.198	[-0.39, 1.89]	0.01
PluralIgnorance	0.48	0.58	50260	0.84	.404	[-0.66, 1.62]	< 0.001
BindingMoral	0.52	0.59	50250	0.89	.374	[-0.64, 1.68]	< 0.001
SystemJust	0.31	0.59	50250	0.53	.596	[-0.85, 1.47]	< 0.001
WorkTogetherNorm	-0.13	0.58	50270	-0.23	.821	[-1.27, 1.01]	< 0.001
NegativeEmotions	-1.31	0.59	50220	-2.21	.027	[-2.47, -0.15]	< 0.001
NegativeEmotions: Ideology	1.74	0.81	50260	2.13	.033	[0.15, 3.33]	< 0.001
SystemJust: Ideology	0.92	0.81	50280	1.14	.255	[-0.67, 2.51]	< 0.001
PsychDistance: Ideology	1.01	0.83	50340	1.22	.224	[-0.62, 2.64]	< 0.001
DynamicNorm: Ideology	0.27	0.81	50300	0.34	.737	[-1.32, 1.86]	< 0.001
FutureSelfCont: Ideology	0.58	0.86	50290	0.68	.498	[-1.11, 2.27]	< 0.001
CollectiveAction: Ideology	0.24	0.81	50250	0.29	.769	[-1.35, 1.83]	< 0.001
BindingMoral: Ideology	0.16	0.81	50270	0.20	.845	[-1.43, 1.75]	< 0.001
WorkTogetherNorm: Ideology	0.01	0.81	50320	0.01	.994	[-1.58, 1.60]	< 0.001
LetterFuture: Ideology	-0.48	0.87	50290	-0.55	.581	[-2.19, 1.23]	< 0.001
PluralIgnorance: Ideology	-0.37	0.81	50280	-0.46	.646	[-1.96, 1.22]	< 0.001
SciConsensus: Ideology	-0.41	0.81	50320	-0.50	.614	[-2.00, 1.18]	< 0.001

Table S9. Results of an ordinal mixed model with number of trees planted in the behavioral task as the dependent variable, condition (11 interventions versus control) as it interacts with self-reported political ideology (median split within each country) as the fixed effects, including by-country random effects. Tests are two-tailed, and no adjustments for multiple comparisons were made.

Fixed Effect	Estimate	SE	*z*	*p*	95% *C.I.*	Cohen's *d*
Ideology	-0.04	0.06	-0.71	.481	[-0.16, 0.08]	0.02
BindingMoral	-0.03	0.06	-0.47	.641	[-0.15, 0.09]	0.02
SciConsens	-0.06	0.06	-1.06	.291	[-0.18, 0.06]	0.04
DynamicNorm	-0.1	0.06	-1.71	.089	[-0.22, 0.02]	0.06
PluralIgnorance	-0.12	0.06	-2.11	.035	[-0.24, -0.002]	0.07
CollectAction	-0.15	0.06	-2.47	.013	[-0.27, -0.03]	0.08
SystemJust	-0.15	0.06	-2.48	.013	[-0.27, -0.03]	0.08
FutureSelfCont	-0.21	0.06	-3.43	<.001	[-0.33, -0.09]	0.11
LetterFuture	-0.32	0.06	-5.18	<.001	[-0.44, -0.20]	0.17
NegativeEmotions	-0.36	0.06	-5.99	<.001	[-0.48, -0.24]	0.20
PsychDistance	-0.37	0.06	-6.09	<.001	[-0.49, -0.25]	0.20
WorkTogetherNorm	-0.37	0.06	-6.40	<.001	[-0.49, -0.25]	0.21
WorkTogetherNorm:Ideology	0.22	0.08	2.66	.008	[0.06, 0.38]	0.11
DynamicNorm:Ideology	0.19	0.08	2.34	.019	[0.03, 0.35]	0.10
FutureSelfCont:Ideology	0.2	0.09	2.29	.022	[0.02, 0.38]	0.11
SciConsens:Ideology	0.17	0.08	2.05	.040	[0.01, 0.33]	0.09
SystemJust:Ideology	0.16	0.08	1.93	.053	[0.003, 0.32]	0.09
PsychDistance:Ideology	0.16	0.08	1.92	.054	[0.003, 0.32]	0.09
LetterFuture:Ideology	0.15	0.09	1.72	.088	[-0.03, 0.33]	0.08
BindingMoral:Ideology	0.12	0.08	1.48	.139	[-0.04, 0.28]	0.07
NegativeEmotions:Ideology	0.11	0.08	1.33	.183	[-0.05, 0.27]	0.06
PluralIgnorance:Ideology	0.09	0.08	1.05	.294	[-0.07, 0.25]	0.05
CollectAction:Ideology	0.08	0.08	0.91	.361	[-0.08, 0.24]	0.04

Table S10. Results from an ordinal mixed model with number of trees planted in the WEPT as the outcome, the interaction of condition and self-reported ideology as the fixed effect, condition time as a fixed effect, with by-country random intercepts. Tests are two-tailed, and no adjustments for multiple comparisons were made.

Fixed Effect	Estimate	SE	*z*	*p*	95% *C.I.*	Cohen's *d*
Time	0.0008	0.00005	17.71	< .001	[0.0007, 0.0009]	< 0.001
Ideology	-0.08	0.06	-1.38	.169	[-0.20, 0.04]	0.05
BindingMoral	0.19	0.06	3.16	.002	[0.07, 0.31]	0.10
SciConsens	0.14	0.06	2.41	.016	[0.02, 0.26]	0.08
PluralIgnorance	0.05	0.06	0.89	.375	[-0.07, 0.17]	0.03
DynamicNorm	0.05	0.06	0.78	.436	[-0.07, 0.17]	0.03
SystemJust	-0.02	0.06	-0.34	.735	[-0.14, 0.10]	0.01
CollectAction	-0.13	0.06	-2.25	.025	[-0.25, -0.01]	0.07
FutureSelfCont	-0.30	0.06	-4.87	< .001	[-0.42, -0.18]	0.16
WorkTogetherNorm	-0.32	0.06	-5.52	< .001	[-0.44, -0.20]	0.18
NegativeEmotions	-0.38	0.06	-6.38	< .001	[-0.50, -0.26]	0.21
PsychDistance	-0.45	0.06	-7.58	< .001	[-0.57, -0.33]	0.25
LetterFutureGen	-0.50	0.06	-8.01	< .001	[-0.62, -0.38]	0.26
DynamicNorm:Ideology	0.25	0.08	3.02	.003	[0.09, 0.41]	0.13
WorkTogetherNorm:Ideology	0.24	0.08	2.98	.003	[0.08, 0.40]	0.13
FutureSelfCont:Ideology	0.23	0.09	2.64	.008	[0.05, 0.41]	0.13
SciConsens:Ideology	0.20	0.08	2.49	.013	[0.04, 0.36]	0.11
SystemJust:Ideology	0.19	0.08	2.29	.022	[0.03, 0.35]	0.10
PsychDistance:Ideology	0.17	0.08	2.08	.038	[0.01, 0.33]	0.09
BindingMoral:Ideology	0.16	0.08	1.93	.053	[0.003, 0.32]	0.09
LetterFutureGen:Ideology	0.16	0.09	1.81	.071	[-0.02, 0.34]	0.09
NegativeEmotions:Ideology	0.14	0.08	1.77	.077	[-0.02 0.30]	0.08
CollectAction:Ideology	0.12	0.08	1.48	.140	[-0.04, 0.28]	0.07
PluralIgnorance:Ideology	0.12	0.08	1.44	.149	[-0.04 0.28]	0.06

Table S11. Results from ordinal mixed model with trees planted in the WEPT as outcome, the interaction of intervention time and condition as the fixed effect, with by-country random intercepts. Tests are two-tailed, and no adjustments for multiple comparisons were made.

Fixed Effect	Estimate	SE	*z*	*p*	95% *C.I.*	Cohen's *d*
Time	0.002	0.0001	10.921	< .001	[0.002, 0.002]	< 0.001
SciConsens	0.449	0.063	7.108	< .001	[0.33, 0.57]	0.25
DynamicNorm	0.428	0.064	6.727	< .001	[0.30 0.55]	0.24
PluralIgnorance	0.411	0.062	6.636	< .001	[0.29, 0.53]	0.23
SystemJust	0.371	0.064	5.821	< .001	[0.25, 0.50]	0.21
CollectAction	0.196	0.066	2.977	.003	[0.07, 0.33]	0.11
WorkTogetherNorm	0.189	0.066	2.858	.004	[0.06, 0.32]	0.10
FutureSelfCont	0.165	0.068	2.414	.016	[0.032, 0.30]	0.09
NegativeEmotions	0.060	0.067	0.897	.370	[-0.07, 0.19]	0.03
LetterFutureGen	-0.021	0.073	-0.285	.775	[-0.16, 0.12]	0.01
PsychDistance	-0.106	0.071	-1.495	.135	[-0.25, 0.03]	0.06
BindingMoral	-0.688	0.062	-11.052	< .001	[-0.81, -0.57]	0.38
BindingMoral:Time	0.804	0.021	37.410	< .001	[0.76, 0.85]	0.43
SciConsens:Time	0.003	0.001	2.748	.006	[0.001, 0.005]	0.002
DynamicNorm: Time	-0.0004	0.0004	-1.202	.229	[-0.001, 0.0004]	< 0.001
PluralIgnorance: Time	-0.001	0.001	-2.539	.011	[-0.003, -0.001]	0.001
SystemJust: Time	-0.001	0.001	-3.914	< .001	[-0.003, -0.001]	0.002
CollectAction: Time	-0.001	0.001	-4.403	< .001	[-0.003, -0.001]	0.002
PsychDistance: Time	-0.001	0.001	-4.910	< .001	[-0.003, -0.001]	0.003
NegativeEmotions: Time	-0.001	0.001	-6.385	< .001	[-0.003, -0.001]	0.004
LetterFutureGen: Time	-0.001	0.001	-6.472	< .001	[-0.003, -0.001]	0.004
FutureSelfCont: Time	-0.001	0.001	-6.501	< .001	[-0.003, -0.001]	0.004
WorkTogetherNorm: Time	-0.002	0.001	-6.571	< .001	[-0.003, -0.001]	0.004

Table S12. Results from exploratory analysis assessing interaction of age and self-reported political ideology on outcome measures in the control condition (climate change beliefs, policy support, and WEPT performance) from linear mixed models (beliefs, policy support) and ordinal mixed model (WEPT) with self-reported ideology and age as fixed effects (mean-centered) allowing these factors to interact, with by-country random intercepts.

Outcome	Ideology:Age Estimate	SE	Test Statistic	*p*	95% *C.I.*	Cohen's *d*
Beliefs	-0.006	0.0001	$t = -6.45$	< .001	[-0.007, -0.006]	0.21
Policy Support	-0.005	0.001	$t = -5.92$	< .001	[-0.007, -0.003]	0.19
WEPT	0.00007	0.0001	$z = 0.82$	.414	[-0.0001, 0.0003]	< 0.001

Table S13. Interventions' effects on self-identified liberals' climate sharing intentions, compared to the control condition. Tests are two-tailed, and no adjustments for multiple comparisons were made.

Fixed Effect .	Estimate	SE	*z*	*p*	95% *C.I.*	Cohen's *d*
(Intercept)	-0.16	0.14	-1.12	.262	[-0.43, 0.11]	0.09
NegativeEmotions	0.65	0.08	8.47	< .001	[0.49, 0.81]	0.37
CollectAction	0.52	0.08	6.65	< .001	[0.36, 0.68]	0.29
FutureSelfCont	0.48	0.08	5.90	< .001	[0.32, 0.64]	0.26
PsychDistance	0.46	0.08	5.82	< .001	[0.30, 0.62]	0.26
LetterFutureGen	0.46	0.08	5.59	< .001	[0.30, 0.62]	0.25
SystemJust	0.37	0.08	4.80	< .001	[0.21, 0.53]	0.21
DynamicNorm	0.32	0.08	4.23	< .001	[0.16, 0.48]	0.19
WorkTogetherNorm	0.32	0.08	4.21	< .001	[0.16, 0.48]	0.19
BindingMoral	0.20	0.08	2.62	< .001	[0.04, 0.36]	0.12
SciConsens	0.19	0.08	2.54	.011	[0.03, 0.35]	0.11
PluralIgnorance	0.19	0.08	2.51	.012	[-0.05, 0.43]	0.11

Table S14. Interventions' effects on self-identified conservatives' climate sharing intentions, compared to the control condition. Tests are two-tailed, and no adjustments for multiple comparisons were made.

Fixed Effect	Estimate	SE	*z*	*p*	95% *C.I.*	Cohen's *d*
(Intercept)	0.31	0.16	1.90	.057	[-0.004, 0.62]	0.17
NegativeEmotions	0.32	0.08	3.94	< .001	[0.16, 0.48]	0.17
CollectAction	0.26	0.08	3.28	< .001	[0.10, 0.42]	0.14
DynamicNorm	0.26	0.08	3.27	.001	[0.10, 0.42]	0.14
PsychDistance	0.22	0.08	2.73	.006	[0.06, 0.38]	0.12
BindingMoral	0.19	0.08	2.38	.017	[0.03, 0.35]	0.10
SystemJust	0.16	0.08	2.02	.043	[0.003, 0.32]	0.09
SciConsens	0.14	0.08	1.76	.078	[-0.02, 0.30]	0.08
WorkTogetherNorm	0.13	0.08	1.72	.085	[-0.03, 0.29]	0.08
FutureSelfCont	0.12	0.08	1.46	.145	[-0.04, 0.28]	0.06
PluralIgnorance	-0.04	0.08	-0.52	.605	[-0.20, 0.12]	0.02

Table S15. Results of a linear mixed effects model with climate sharing intentions as the dependent variable, condition (11 interventions versus control) as it interacts with self-reported political ideology (median split within each country) as the fixed effects, including by-participant and by-country random effects.

Fixed Effect	Estimate	SE	*z*	*p*	95% *C.I.*	Cohen's *d*
(Intercept)	0.27	0.15	1.79	.073	[-0.02, 0.56]	0.15
Ideology	-0.42	0.08	-5.36	< .001	[-0.58, -0.26]	0.24
LetterFutureGen	0.34	0.08	4.04	< .001	[0.18, 0.50]	0.18
NegativeEmotions	0.31	0.08	3.95	< .001	[0.15, 0.47]	0.17
CollectAction	0.26	0.08	3.27	.001	[0.10, 0.42]	0.14
DynamicNorm	0.25	0.08	3.27	.001	[0.09, 0.41]	0.14
PsychDistance	0.23	0.08	2.86	.004	[0.07, 0.39]	0.13
BindingMoral	0.18	0.08	2.36	.018	[0.02, 0.34]	0.10
SystemJust	0.15	0.08	1.93	.053	[-0.01, 0.31]	0.09
SciConsens	0.14	0.08	1.78	.075	[-0.02, 0.30]	0.08
WorkTogetherNorm	0.14	0.08	1.78	.076	[-0.02, 0.30]	0.08
FutureSelfCont	0.11	0.08	1.40	.161	[-0.05, 0.27]	0.06
PluralIgnorance	-0.04	0.08	-0.49	.626	[-0.20, 0.12]	0.02
FutureSelfCont:Ideology	0.39	0.11	3.32	< .001	[0.17, 0.61]	0.20
NegativeEmotions:Ideology	0.35	0.11	3.17	.002	[0.13, 0.57]	0.19
CollectAction:Ideology	0.28	0.11	2.54	.011	[0.06, 0.50]	0.15
PluralIgnorance:Ideology	0.24	0.11	2.22	.026	[0.02, 0.46]	0.13
PsychDistance:Ideology	0.24	0.11	2.10	.036	[0.02, 0.46]	0.13
SystemJust:Ideology	0.23	0.11	2.09	.037	[0.01, 0.45]	0.13
WorkTogetherNorm:Ideology	0.19	0.11	1.80	.072	[-0.03, 0.41]	0.11
LetterFutureGen:Ideology	0.13	0.12	1.12	.260	[-0.11, 0.37]	0.07
DynamicNorm:Ideology	0.09	0.11	0.81	.421	[-0.13, 0.31]	0.05
SciConsens:Ideology	0.07	0.11	0.69	.492	[-0.15, 0.29]	0.04
BindingMoral:Ideology	0.02	0.11	0.21	.831	[-0.20, 0.24]	0.01

Table S16. Results from ordinal mixed model with trees planted in the WEPT as outcome, the interaction of intervention time, condition, and self-reported political ideology (median split within each country) as the fixed effect, with by-country random intercepts. Tests are two-tailed, and no adjustments for multiple comparisons were made.

Fixed Effect	Estimate	SE	*z*	*p*	95% *C.I.*	Cohen's *d*
Time	0.002	0.001	5.88	< .001	[0.002, 0.002]	0.003
Ideology	-0.096	0.132	-0.73	.465	[-0.35, 0.16]	0.05
PluralIgnorance	0.413	0.106	3.92	< .001	[0.21, 0.62]	0.23
DynamicNorm	0.369	0.109	3.39	< .001	[0.16, 0.58]	0.20
SystemJust	0.212	0.113	1.87	.061	[-0.01, 0.43]	0.12
CollectAction	0.167	0.115	1.45	.147	[-0.06, 0.39]	0.09
FutureSelfCont	0.140	0.116	1.20	.230	[-0.09, 0.37]	0.08
WorkTogetherNorm	0.105	0.112	0.94	.349	[-0.11, 0.32]	0.06
SciConsens	0.110	0.119	0.92	.357	[-0.12, 0.34]	0.06
NegativeEmotions	0.073	0.114	0.64	.524	[-0.15, 0.30]	0.04
LetterFutureGen	-0.125	0.124	-1.01	.313	[-0.37, 0.12]	0.07
PsychDistance	-0.261	0.122	-2.14	.033	[-0.50, -0.02]	0.14
BindingMoral	-0.757	0.109	-6.94	< .001	[-0.97, -0.54]	0.42
BindingMoral: Time	0.833	0.034	24.46	< .001	[0.77, 0.90]	0.04
SciConsens: Time	0.018	0.003	5.37	< .001	[0.012, 0.024]	0.01
SystemJust: Time	-0.001	0.001	-1.17	.243	[-0.003, 0.001]	< 0.001
PsychDistance: Time	-0.001	0.001	-2.45	.014	[-0.001, -0.001]	0.001
DynamicNorm: Time	-0.001	0.001	-2.53	.011	[-0.003, 0.001]	0.001
CollectAction: Time	-0.001	0.001	-2.90	.004	[-0.001, -0.001]	0.002
LetterFutureGen: Time	-0.002	0.001	-3.73	< .001	[-0.002, -0.002]	0.002
FutureSelfCont: Time	-0.002	0.001	-4.28	< .001	[-0.002, -0.002]	0.001
PluralIgnorance: Time	-0.002	0.001	-4.39	< .001	[-0.002, -0.002]	0.002
WorkTogetherNorm: Time	-0.002	0.001	-4.44	< .001	[-0.002, -0.002]	0.002
NegativeEmotions: Time	-0.002	0.001	-4.50	< .001	[-0.002, -0.002]	0.002
SciConsens: Ideology	0.573	0.156	3.68	< .001	[0.27, 0.88]	0.32
PsychDistance: Ideology	0.369	0.168	2.20	.028	[0.04, 0.70]	0.20
SystemJust: Ideology	0.317	0.154	2.05	.040	[0.02, 0.62]	0.17

Fixed Effect	Estimate	SE	*z*	*p*	95% *C.I.*	Cohen's *d*
CollectAction: Ideology	0.213	0.157	1.36	.175	[-0.09, 0.52]	0.02
LetterFutureGen: Ideology	0.221	0.171	1.30	.195	[-0.11, 0.56]	0.02
DynamicNorm: Ideology	0.174	0.153	1.14	.254	[-0.13, 0.47]	0.01
FutureSelfCont: Ideology	0.138	0.163	0.84	.400	[-0.18, 0.46]	0.01
BindingMoral: Ideology	0.123	0.150	0.82	.414	[-0.17, 0.42]	0.01
NegativeEmotions: Ideology	0.066	0.158	0.42	.677	[-0.24, 0.38]	0.01
PluralIgnorance: Ideology	-0.031	0.150	-0.21	.837	[-0.33, 0.26]	0.003
Time: Ideology	0.000	0.001	-0.22	.824	[-0.001, 0.002]	< 0.001
WorkTogetherNorm: Ideology	-0.040	0.162	-0.25	.807	[-0.36, 0.28]	0.004
PluralIgnorance: Time: Ideology	0.003	0.001	3.16	.002	[0.001, 0.005]	< 0.001
WorkTogetherNorm: Time: Ideology	0.002	0.001	2.47	.013	[0.001, 0.004]	0.001
DynamicNorm: Time: Ideology	0.001	0.001	1.53	.126	[-0.001, 0.003]	< 0.001
BindingMoral: Time: Ideology	0.039	0.049	0.80	.421	[-0.06, 0.14]	0.001
NegativeEmotions: Time: Ideology	0.000	0.001	0.79	.430	[-0.002, 0.002]	< 0.001
FutureSelfCont: Time: Ideology	0.000	0.001	0.74	.458	[-0.002, 0.002]	< 0.001
LetterFutureGen: Time: Ideology	0.000	0.001	0.02	.987	[-0.002, 0.002]	< 0.001
CollectAction: Time: Ideology	0.000	0.001	-0.42	.672	[-0.002, 0.002]	< 0.001
PsychDistance: Time: Ideology	0.000	0.001	-0.73	.468	[-0.002, 0.002]	< 0.001
SystemJust: Time: Ideology	-0.001	0.001	-1.22	.224	[-0.003, 0.002]	< 0.001
SciConsens: Time: Ideology	-0.019	0.003	-5.44	< .001	[-0.02, -0.01]	0.01

International Ethics Review Statement

Collaborators on each research team, in each country, acquired individual IRB approval by ethics review committees before data collection began. The table below lists the countries, and the names of the review boards who granted approval. The full list of all ethics boards approvals can also be found on OSF: https://osf.io/ytf89/

Table S17. List of Review Boards that approved data collection for this work.

Country Team	Name of Review Board
Algeria	Aarhus University's Research Ethics Committee
Armenia	RUHR-UNIVERSITÄT BOCHUM Fakultät für Psychologie Ethikkommission
Australia	Research Ethics Office, The Australian National University
Austria	The Ethics Committee of the Faculty of Business, Economics and Social Sciences of the University of Bern
Belgium	Comité d'Avis Ethique de la Faculté des Sciences Psychologiques et de l'Education, Université Libre de Bruxelles
Belgium	Sociaal-Maatschappelijke Ethische Commissie (SMEC), KU Leuven
Brazil	COMISSÃO NACIONAL DE ÉTICA EM PESQUISA
Bulgaria	The Ethics Committee of the Faculty of Business, Economics and Social Sciences of the University of Bern
Canada	The University of British Columbia, Office of Research Services, Behavioural Research Ethics Board
Canada	Simon Frazer University Research Ethics
Czechia	IRB, Department of Management, Prague University of Economics and Business
Chile	Comité Ético Científico de Ciencias Sociales, Pontificia Universidad Católica de Chile
China (TW)	Northwestern University, Institutional Review Board
Denmark	The Research Ethics Commitee, Aarhus BSS
Ecuador	University of Oslo, Faculty of Social Sciences, Department of Psychology's Research Ethics Committee
Finland	IRB Universitetet i Stavanger, Det samfunnsvitenskapelige fakultet/ Institutt for sosialfag
France	CUREG, Université de Genéve
Gambia	Ethikkommission Paris-Lodron-Universität Salzburg
Germany	The Ethics Committee of the Faculty of Business, Economics and Social Sciences of the University of Bern
Ghana	Ethikkommission der Fakultät für Mathematik, Informatik und Statistik, LMU München
Greece	Univeristy of Crete Research Ethics Committee
India	Ethics Review Board (FMG-UvA), University of Amsterdam

Ireland	EHS Research Ethics, University of Limerick
Israel	Social Sciences Ethics Committee, The Hebrew Univesristy of Jerusalem
Italy	Institutional Review Board for Social & Behavioral Sciences, University of Virginia
Italy	COMITATO ETICO DELLA RICERCA PSICOLOGICA, Dipartimenti/Sezione di Psicologia, Università di Padova
Japan	IRB of the Kochi University of Technology
Japan	The Ethics Review Committee on Research with Human Subjects of Waseda University
Latvia	The IRB of the Latvijas Universitātes Humanitāro
Mexico	Research Commission of the Leuphana University of Lüneburg
Morocco	Institutional Review Board at NYU Abu Dhabi
Netherlands	Ethics Review Board, Tillburg School of Social and Behavioral Sciences, Tilburg University
Netherlands	Ethics Review Board, Communication Science, University of Amsterdam
Netherlands	Ethics Review Board, Communication Science, University of Amsterdam
New Zealand	the Science & Med DERC Chair at the Australian National University
Nigeria	Science, Technology, Engineering, and Mathematics Ethical Review Committee at the University of Birmingham
North Macedonia	Етичкиот поткомитет за медицина, фармација, ветерина и стоматологија при МАНУ, Македонска академија на науките и уметностите
Norway	Norwegian School of Economics Institutional Review Board
Peru	Universidad Peruana Cayetano Heredia
Philippines	University of the Philippines Visayas RESEARCH ETHICS BOARD
Poland	Faculty of Philosophy and Social Sciences, Nicolaus Copernicus University
Poland	Faculty of Philosophy and Social Sciences, Nicolaus Copernicus University
Portugal	the Ethical and Deontological Committee for Scientific Research (CEDIC) at the University of Lusofona
Romania	the Institutional Review Board at the University of Pennsylvania
Russia	HSE University, Center for sociocultural research
Russia	Ethics committee of the Ural Federal University
Russia	Ethics Committee of The South Ural University of Technology
Saudi Arabia	Research Committee at the Canadian University Dubai
Serbia	Ethics Committee of the Department of Psychology, Faculty of Philosophy, University of Novi Sad

Slovakia	Univerzita Komenskeho v Bratislave
Slovenia	The Ethics Committee of the Faculty of Business, Economics and Social Sciences of the University of Bern
South Africa	The Ethics Committee of the Faculty of Psychology of the University of Basel
South Korea	Ulsan National Institute of Science and Technology
Spain	Comite Etico de Investigacion con Humanos Universidad de Cordoba
Spain	The Committee for the Use of Human Subjects in Research (CUHSR) at Esade
Sri Lanka	Research Ethics at The London School of Economics and Political Sciences
Sudan	Institutional Review Board at NYU Abu Dhabi
Sweden	IRB exempt. Waiver added to folder.
Switzerland	The Ethics Committee of the Faculty of Business, Economics and Social Sciences of the University of Bern
Switzerland	Research Ethics Commission of the University of Lausanne (CER-UNIL)
Taiwan	College of Management, National Kaohsiung University of Science and Technology
Thailand	School of Global Studies, Thammasat University
Turkey	Human Research Ethics Committee of Kadir Has University
Turkey	Human Research Ethics Committee of Kadir Has University
United Arab Emirates	Institutional Review Board at NYU Abu Dhabi
Uganda	The Science-Geosciences Ethics Review Board (SG ERB) at Utrecht University
United Kingdom	The University of Birmingham's research ethics processes
United Kingdom	Science, Technology, Engineering, and Mathematics Ethical Review Committee at the University of Birmingham
United Kingdom	The School Research Ethics Panel
United Kingdom	The University of Birmingham's research ethics processes
Ukraine	The ethical review board at Kyiv School of Economics
Uruguay	Comite de Etica en Investigacion de la Facultad de Psicologia de la Universidad de la Republica
United States	Science, Technology, Engineering, and Mathematics Ethical Review Committee at the University of Birmingham
United States	Stanford Research Compliance Office
United States	The internal review board at New York University

Venezuela	FSW Research Ethics Review at the University of Amsterdam
Vietnam	University of Economics Ho Chi Minh City
