# Supplementary material for: The differential impact of climate interventions along the political divide in 60 countries
Source: Nat Commun. 2024 May 8;15:3885. doi: 10.1038/s41467-024-48112-8 (PMC11078920; doi:10.1038/s41467-024-48112-8)
Supplement: Supplementary file 3 — Reporting Summary [file 41467_2024_48112_MOESM3_ESM.pdf]

nature portfolio

Corresponding author(s): Madalina Vlasceanu

Last updated by author(s): 2024/03/20

Reporting Summary

Nature Portfolio wishes to improve the reproducibility of the work that we publish. This form provides structure for consistency and transparency in reporting. For further information on Nature Portfolio policies, see our Editorial Policies and the Editorial Policy Checklist.

Statistics

For all statistical analyses, confirm that the following items are present in the figure legend, table legend, main text, or Methods section.

n/a	Confirmed	
☐	☒	The exact sample size (*n*) for each experimental group/condition, given as a discrete number and unit of measurement
☐	☒	A statement on whether measurements were taken from distinct samples or whether the same sample was measured repeatedly
☐	☒	The statistical test(s) used AND whether they are one- or two-sided<br>*Only common tests should be described solely by name; describe more complex techniques in the Methods section.*
☐	☒	A description of all covariates tested
☐	☒	A description of any assumptions or corrections, such as tests of normality and adjustment for multiple comparisons
☐	☒	A full description of the statistical parameters including central tendency (e.g. means) or other basic estimates (e.g. regression coefficient) AND variation (e.g. standard deviation) or associated estimates of uncertainty (e.g. confidence intervals)
☐	☒	For null hypothesis testing, the test statistic (e.g. *F*, *t*, *r*) with confidence intervals, effect sizes, degrees of freedom and *P* value noted<br>*Give P values as exact values whenever suitable.*
☒	☐	For Bayesian analysis, information on the choice of priors and Markov chain Monte Carlo settings
☐	☒	For hierarchical and complex designs, identification of the appropriate level for tests and full reporting of outcomes
☐	☒	Estimates of effect sizes (e.g. Cohen's *d*, Pearson's *r*), indicating how they were calculated

Our web collection on statistics for biologists contains articles on many of the points above.

Software and code

Policy information about availability of computer code

Data collection	The data were collected through the Qualtrics platform. Data can be accessed on OSF: https://osf.io/ytf89/
Data analysis	Data analysis was performed using R (V 4.3.2). Figure creation and data cleaning was performed in Python (V 3.9.1). All analyses code can be found on github: https://github.com/mvlasceanu/climatepolarization. Specific R packages for analyses include lme4, ordinal, and BayesFactor.

For manuscripts utilizing custom algorithms or software that are central to the research but not yet described in published literature, software must be made available to editors and reviewers. We strongly encourage code deposition in a community repository (e.g. GitHub). See the Nature Portfolio guidelines for submitting code & software for further information.

Data

Policy information about availability of data

All manuscripts must include a data availability statement. This statement should provide the following information, where applicable:

- Accession codes, unique identifiers, or web links for publicly available datasets
- A description of any restrictions on data availability
- For clinical datasets or third party data, please ensure that the statement adheres to our policy

All data can be found on OSF: https://osf.io/ytf89/

Research involving human participants, their data, or biological material

Policy information about studies with human participants or human data. See also policy information about sex, gender (identity/presentation), and sexual orientation and race, ethnicity and racism.

Reporting on sex and gender	Participants were asked to report their identified gender. The research teams did not ask participants to report their sex. Regarding participants' self-reported gender: 24,883 identified as male, 25,765 identified as female, 350 identified as non-binary, and 226 preferred not to state their gender. Based on our sample sizes, our findings apply to multiple genders. Sex and/or gender were not central to the research questions, but the study and items were designed in a way as to be gender-inclusive. Although gender and/or sex were not theoretically central to our research aims, we included analyses that included gender as a covariate to statistically adjust for potential effects of participants' gender identities.
Reporting on race, ethnicity, or other socially relevant groupings	The study asked participants to report their age, gender, income, and education level. All measures were self-reported. Age was reported in years. Options for gender included: male, female, and non-binary. Education level was reported in years of formal education completed. Income was reported as "total yearly family/household income" ranging from less than $10,000 to over $200,000. Research teams did not ask participants' to report on race or ethnicity. These variables were used as covariates in analyses to statistically adjust fro the effects of these demographic variables.
Population characteristics	See above.
Recruitment	Participants (N=51,224, from 60 counties, were mostly recruited through online data collection platforms (80.8%) or via convenience/snowball sampling (19.1%). They were randomly assigned to one of 11 experimental conditions, or a no-intervention control condition. All research teams were given information on how to translate and adapt the study to each country. As with any Online study effort, the reachable population is restricted to those who have access to a computer with an Internet connection, as well as those interested in taking part in research. This selection bias could potentially limit the generalizability of the results, although we feel confident the results are buffered against this given the overall sample size and scope of the countries that were included.
Ethics oversight	Ethics approval was obtained independently by each research team from the respective Institutional Review Board (IRB) associated with their institution. Analyses only included datasets submitted along with IRB approval. The individual review boards are listed in full in the supplemental materials with the manuscript. With 60 countries (and some countries involving multiple research teams and thus multiple review boards, the list is substantial).<br><br>We paste the list below:<br>Aarhus University's Research Ethics Committee<br>RUHR-UNIVERSITÄT BOCHUM Fakultät für Psychologie Ethikkommission<br>Research Ethics Office, The Australian National University<br>The Ethics Committee of the Faculty of Business, Economics and Social Sciences of the University of Bern<br>Comité d'Avis Ethique de la Faculté des Sciences Psychologiques et de l'Education, Université Libre de Bruxelles<br>Sociaal-Maatschappelijke Ethische Commissie (SMEC), KU Leuven<br>COMISSÃO NACIONAL DE ÉTICA EM PESQUISA<br>The Ethics Committee of the Faculty of Business, Economics and Social Sciences of the University of Bern<br>The University of British Columbia, Office of Research Services, Behavioural Research Ethics Board<br>Simon Frazer University Research Ethics<br>IRB, Department of Management, Prague University of Economics and Business<br>Comité Ético Científico de Ciencias Sociales, Pontificia Universidad Católica de Chile<br>Northwestern University, Institutional Review Board<br>The Research Ethics Commitee, Aarhus BSS<br>University of Oslo, Faculty of Social Sciences, Department of Psychology's Research Ethics Committee<br>IRB Universitetet i Stavanger, Det samfunnsvitenskapelige fakultet/ Institutt for sosialfag<br>CUREG, Université de Genéve<br>Ethikkommission Paris-Lodron-Universität Salzburg<br>The Ethics Committee of the Faculty of Business, Economics and Social Sciences of the University of Bern<br>Ethikkommission der Fakultät für Mathematik, Informatik und Statistik, LMU München<br>Univeristy of Crete Research Ethics Committee<br>Ethics Review Board (FMG-UvA), University of Amsterdam<br>EHS Research Ethics, University of Limerick<br>Social Sciences Ethics Committee, The Hebrew Univesristy of Jerusalem<br>Institutional Review Board for Social & Behavioral Sciences, University of Virginia<br>COMITATO ETICO DELLA RICERCA PSICOLOGICA, Dipartimenti/Sezione di Psicologia, Università di Padova<br>IRB of the Kochi University of Technology<br>The Ethics Review Committee on Research with Human Subjects of Waseda University<br>The IRB of the Latvijas Universitātes Humanitāro<br>Research Commission of the Leuphana University of Lüneburg<br>Institutional Review Board at NYU Abu Dhabi<br>Ethics Review Board, Tillburg School of Social and Behavioral Sciences, Tilburg University<br>Ethics Review Board, Communication Science, University of Amsterdam<br>the Science & Med DERC Chair at the Australian National University<br>Science, Technology, Engineering, and Mathematics Ethical Review Committee at the University of Birmingham<br>Етичкиот поткомитет за медицина, фармација, ветерина и стоматологија при МАНУ, Македонска академија на науките и уметностите<br>Norwegian School of Economics Institutional Review Board<br>Universidad Peruana Cayetano Heredia

University of the Philippines Visayas RESEARCH ETHICS BOARD
Faculty of Philosophy and Social Sciences, Nicolaus Copernicus University
the Ethical and Deontological Committee for Scientific Research (CEDIC) at the University of Lusofona
the Institutional Review Board at the University of Pennsylvania
HSE University, Center for sociocultural research
Ethics committee of the Ural Federal University
Ethics Committee of The South Ural University of Technology
Research Committee at the Canadian University Dubai
Ethics Committee of the Department of Psychology, Faculty of Philosophy, University of Novi Sad
Univerzita Komenskeho v Bratislave
The Ethics Committee of the Faculty of Business, Economics and Social Sciences of the University of Bern
The Ethics Committee of the Faculty of Psychology of the University of Basel
Ulsan National Institute of Science and Technology
Comite Etico de Investigacion con Humanos Universidad de Cordoba
The Committee for the Use of Human Subjects in Research (CUHSR) at Esade
Research Ethics at The London School of Economics and Political Sciences
Institutional Review Board at NYU Abu Dhabi
The Ethics Committee of the Faculty of Business, Economics and Social Sciences of the University of Bern
Research Ethics Commission of the University of Lausanne (CER-UNIL)
College of Management, National Kaohsiung University of Science and Technology
School of Global Studies, Thammasat University
Human Research Ethics Committee of Kadir Has University
Institutional Review Board at NYU Abu Dhabi
The Science-Geosciences Ethics Review Board (SG ERB) at Utrecht University
The University of Birmingham's research ethics processes
Science, Technology, Engineering, and Mathematics Ethical Review Committee at the University of Birmingham
The School Research Ethics Panel
The University of Birmingham's research ethics processes
The ethical review board at Kyiv School of Economics
Comite de Etica en Investigacion de la Facultad de Psicologia de la Universidad de la Republica
Science, Technology, Engineering, and Mathematics Ethical Review Committee at the University of Birmingham
Stanford Research Compliance Office
The internal review board at New York University
FSW Research Ethics Review at the University of Amsterdam
University of Economics Ho Chi Minh City

Note that full information on the approval of the study protocol must also be provided in the manuscript.

Field-specific reporting

Please select the one below that is the best fit for your research. If you are not sure, read the appropriate sections before making your selection.

☐ Life sciences ☒ Behavioural & social sciences ☐ Ecological, evolutionary & environmental sciences

For a reference copy of the document with all sections, see nature.com/documents/nr-reporting-summary-flat.pdf

Behavioural & social sciences study design

All studies must disclose on these points even when the disclosure is negative.

Study description	This study employs a quantitative, experimental design.
Research sample	The sample consisted of 51,224 adult participants recruited from 60 countries across the world. 50% of the sample identified as female, 49% identified as male, and 1% identified as non-binary. The mean age of the sample was 39.6 years. This sample was chosen to encapsulate as broad and inclusive of a collection as possible, and thus is highly representative. In 41 of the 60 countries, the samples recruited were census-matched to the countries' populations on at least one of the following: age, gender, education level, region, ethnicity, and income. No statistical method was used to determine sample size, individual research teams were encouraged to recruit large sample sizes that matched country-level demographic qualities.
Sampling strategy	Participants (N=51,224, from 60 counties, with 68% of the countries including samples representative on at least one demographic variable) were mostly recruited through online data collection platforms (80.8%) or via convenience/snowball sampling (19.1%). The organizational team submitted a call for collaboration on social media and by posting on various mailing lists. We asked researchers from around the world to join our project. Collaborators willing to collect data were asked to collect 500 responses, which was determined to be a sufficient sample size per country to gain a representative sample and achieve conventional levels of power based on similar intervention-based studies.
Data collection	After confirming collaborators' contributions, we contacted the collaborators whose interventions had been selected to be included in the main study, to coordinate the intervention implementation and programming on the Qualtrics survey platform (https://www.qualtrics.com/). After finalizing analysis plans and pre-registering the study, we sent our collaborators the final version of the study in Qualtrics along with an in-depth instructions manual (available here: https://osf.io/e452d?view_only=180bc6fc47a8421cae6f2eea8978b222) on how to translate and adapt the study to each country. We also instructed our collaborators to obtain ethics approval from their institutions' review boards before launching data collection. All collaborators were

	given 8 months (until March 2023) to submit their data. Participants completed the study alone at a computer. Experimenters were blind to conditions/hypotheses.
Timing	Data were collected from July 2022 through April 2023
Data exclusions	A total of 83,927 people completed the study. Of them, 51,224 participants from 60 countries who passed the attention checks were included in the analyses (32,703 excluded). This exclusion rule was pre-established before data analyses began.
Non-participation	Research teams were not asked to report how many participants dropped out of the studies.
Randomization	Participants were randomly assigned to either the control group or one of eleven intervention groups.

Reporting for specific materials, systems and methods

We require information from authors about some types of materials, experimental systems and methods used in many studies. Here, indicate whether each material, system or method listed is relevant to your study. If you are not sure if a list item applies to your research, read the appropriate section before selecting a response.

Materials & experimental systems

n/a	Involved in the study
☒	☐ Antibodies
☒	☐ Eukaryotic cell lines
☒	☐ Palaeontology and archaeology
☒	☐ Animals and other organisms
☒	☐ Clinical data
☒	☐ Dual use research of concern
☒	☐ Plants

Methods

n/a	Involved in the study
☒	☐ ChIP-seq
☒	☐ Flow cytometry
☒	☐ MRI-based neuroimaging
